# Share – Workpackage 5: evidence based recommendations for diagnosis and treatment of juvenile idiopathic arthritis

**DOI:** 10.1186/1546-0096-12-S1-P171

**Published:** 2014-09-17

**Authors:** Sebastiaan Vastert, Victor Boom, Angelo Ravelli, Alberto Martini, Helen Foster, Nico Wulffraat, Jordi Anton, Tamas Constantin, Pavla Dolezalova, Gerd Horneff, Pekka Lahdenne, Bo Magnussen, Kirsten Minden, Kiran Nistala, Pierre Quartier, Ingrida Rumbla, Nicola Ruperto, Vanessa Remy Piccolo, Ricardo Russo, Sefi Uziel, C Wouters

**Affiliations:** 1 Pediatric Rheumatology, University Medical Center , Utrecht, Netherlands; 2 Pediatric Rheumatology, G Gaslini Institute, Genua, Italy; 3 Pediatric Rheumatology, The Medical School, Newcastle University, Newcastle, UK

## Introduction

Juvenile Idiopathic Arthritis (JIA) is one of the most common chronic pediatric rheumatic diseases (PRD). As is the case for most PRD's, evidence-based guidelines are sparse and management is based to great extent on physician’s experience. Moreover, there are differences between nations regarding availability and financing of biological therapies. Consequently, treatment regimens differ throughout Europe. In 2012, a European initiative called SHARE (Single Hub and Access point for pediatric Rheumatology in Europe) was launched to optimize and disseminate diagnostic and management regimens in Europe for children and young adults with rheumatic diseases.

## Objectives

To provide evidence based recommendations for diagnosis and treatment of JIA.

## Methods

Evidence based recommendations were developed using the European League Against Rheumatism (EULAR) standard operating procedure [1]. An expert committee was instituted, consisting of pediatric rheumatologists from across Europe with expertise in JIA. The expert committee defined search terms for the systematic literature review. Two independent experts scored articles for validity and level of evidence. Recommendations derived from the literature were evaluated by an online survey. Those with less than 80% agreement during the online survey were reformulated. Subsequently, all recommendations were discussed by the experts at a consensus meeting using the nominal group technique [2]. Recommendations were accepted if more than 80% agreement was reached.

## Results

The literature search yielded 4723 articles, of which 174 were considered relevant. The included articles were scored for validity and level of evidence. Recommendations were formulated based on the valid papers and were discussed and adjusted where needed during the consensus meeting. In total, 10 recommendations for diagnosis and 31 for treatment were accepted with more than 80% agreement. Topics covered for diagnosis and for treatment are shown in Table 1.

**Table 1:** 

Juvenile idiopathic arthritis	
**Diagnosis**	**Treatment**

The value of MRI in the diagnosis arthritis	Steroids (locally and systemically administered)

The value of ultrasound in the diagnosis of arthritis	DMARDS

Biomarkers for diagnosis of JIA	Biologicals

Diagnosis of complications	Treatment of complications

## Conclusion

The SHARE initiative provides recommendations for diagnosis and treatment of JIA and thereby facilitates improvement and uniformity of care throughout Europe. In the subsequent phase of the project, best practices identified from literature will be completed with the ‘experts opinion’ in order to formulate diagnostic and management guidelines as best practices for care of JIA patients throughout Europe.

## Disclosure of interest

S. Vastert Consultant for: Novartis, V. Boom: None Declared, A. Ravelli: None Declared, A. Martini: None Declared, H. Foster: None Declared, N. Wulffraat Grant / Research Support from: Abbvie, GSK, Roche, Consultant for: Novartis, Genzyme, Roche, Pfizer.
